# Beyond trial types

**DOI:** 10.1007/s00426-014-0570-8

**Published:** 2014-05-04

**Authors:** Mads Dyrholm, Signe Vangkilde, Claus Bundesen

**Affiliations:** Center for Visual Cognition, University of Copenhagen, Øster Farimagsgade 2A, 1353 Copenhagen, Denmark

## Abstract

Conventional wisdom on psychological experiments has held that when one or more independent variables are manipulated it is essential that all other conditions are kept constant such that confounding factors can be assumed negligible (Woodworth, 1938). In practice, the latter assumption is often questionable because it is generally difficult to guarantee that all other conditions are constant between any two trials. Therefore, the most common way to check for confounding violations of this assumption is to split the experimental conditions in terms of “trial types” to simulate a reduction of unintended trial-by-trial variation. Here, we pose a method which is more general than the use of trial types: use of mathematical models treating measures of potentially confounding factors and manipulated variables as equals on the single-trial level. We show how the method can be applied with models that subsume under the generalized linear item response theory (GLIRT), which is the case for most of the well-known psychometric models (Mellenbergh, 1994). As an example, we provide a new analysis of a single-letter recognition experiment using a nested likelihood ratio test that treats manipulated and measured variables equally (i.e., in exactly the same way) on the single-trial level. The test detects a confounding interaction with time-on-task as a single-trial measure and yields a substantially better estimate of the effect size of the main manipulation compared with an analysis made in terms of trial types.

## Beyond trial types

Common wisdom has implied a restrictive conception of psychological experiments. In the words of one of the fathers of modern experimental psychology, Robert S. Woodworth, “an experimenter is said to control the conditions in which an event occurs” (Woodworth, 1938). By manipulating the experimental conditions (changing trial types), one or more independent variables are varied, and the associated variations in the participants’ performance or reported experience (the dependent variables) are observed. According to Woodworth, “whether one or more independent variables are used, it remains essential that all other conditions be constant. Otherwise you cannot connect the effect observed with any definite cause” (Woodworth, 1938).

Notwithstanding this claim, cognitive neuroscientists have recently begun to use physiological measures that fluctuate from trial to trial as explanatory variables along with manipulated variables (see Cavanagh et al., 2011; O’Doherty, Hampton, & Kim, 2007). We further this development by proposing that using mathematical models, single-trial measures and manipulated variables can be treated as equals in statistical tests. The method is readily applicable to models that subsume under the generalized linear item response theory (GLIRT), which is the case for most of the well-known psychometric models (Mellenbergh, 1994). In GLIRT, a linear combination of latent and observed explanatory variables is used as a predictor of the expected response of a participant to a stimulus item in a specified format. We show that the special case of the Theory of Visual Attention (TVA; Bundesen, 1990) used for modeling single-stimulus recognition (e.g., Bundesen & Harms, 1999; Vangkilde, Coull, & Bundesen, 2012) is also a special case of GLIRT, and we present a new analysis of a single-letter recognition experiment based on this theory (Vangkilde et al., 2012, Experiment 3). The new analysis shows that the expected response of a participant on a particular trial depends strongly on the time-on-task associated with the trial in question. This confound is grossly underestimated by a traditional analysis in terms of trial types (early vs. late trials), and it even goes undetected in a standard post hoc check.^1^

## Single-letter recognition under GLIRT

TVA is often used to describe an observer’s recognition accuracy as a function of exposure duration *t*. In its most commonly applied form, TVA provides estimates for the following perceptual parameters: visual short-term memory (VSTM) capacity *K* (in units of elements), processing speed *C* (rate of categorization in units of elements per second), a temporal threshold *t*_0_ (seconds), attentional weights {*w*_*x*_} (unitless) for a fixed set of display positions {*x*}, and a measure of the efficiency of top-down control α (unitless ratio of the attentional weight of a distractor to the weight of a target). This particular parameterization has been widely applied in studies of partial report, whole report, and change detection (Bundesen & Habekost, 2008; Duncan et al., 1999; Gillebert et al., 2012; Habekost & Starrfelt, 2009; Hung, Driver & Walsh, 2005, 2011; Kyllingsbæk & Bundesen, 2009; Shibuya & Bundesen, 1988). The parameters have traditionally been assumed to be nearly constant within each trial type (Kyllingsbæk, 2006), but recent advances have shown that this assumption leads to systematic errors (Dyrholm, Kyllingsbæk, Espeseth & Bundesen, 2011). Here, we estimate parameters on individual trials (the *v* values in Eqs. 1 and 2 below on every trial *n*) using a linear predictor (the right-hand side of Eq. 2) that varies between any two trials (for related work on single-trial inference using the number of correctly reported targets on a given trial for inferring the number of distractors in VSTM on the same trial of a partial report task, see Dyrholm, Kyllingsbæk, Vangkilde et al. 2011).

Consider a single-stimulus recognition task in which participants are instructed to report the identity of a single target followed by a mask. The delay between the target and its mask defines the target exposure duration, which enters TVA as the variable *t*. Summed across *N* Bernoulli trials with the same exposure duration *t*, the number of correct responses follows a binomial distribution with parameters *N* and *p*, where the probability *p* that a given item is correctly reported defines the expected value of the participant’s response on each trial (Mellenbergh, 1994). In the single-stimulus case (Bundesen & Harms, 1999; Dyrholm, Kyllingsbæk, Espeseth & Bundesen, 2011), TVA implies that *p* = 1 − exp(− τ*v*) where τ = *t* − *t*_0_ is the effective exposure duration if *t* exceeds the temporal threshold *t*_0_, whereas *p* = 0 if *t* ≤ *t*_0_. The parameter *v* is the conventional single-stimulus equivalent of the *C* parameter of TVA. From this, we derive a function of the expected item response *p* on a given trial.

1 $$v_{n} = - \ln(1 - p_{n} )/\tau_{n}$$

where the subscript *n* is the trial number. This function is monotonic and differentiable as required for a link function under GLIRT. Inserting a linear predictor of the logarithm of *v*_*n*_,

2 $$\ln\left( {v_{n} } \right) \, = a_{1} x_{1n} + a_{2} x_{2n} + \, . \, . \, . \, + a_{M} x_{Mn}$$

we obtain a model of single-stimulus recognition that satisfies sufficient requirements to be subsumed under GLIRT^2^: The responses are modeled as independently distributed across trials given the values of the explanatory variables; a distribution of the responses occurs according to the given item format (here a dichotomous format: correct vs. incorrect); and the item responses *p*_*n*_ are explained by a continuous latent variable *v*_*n*_ (Mellenbergh, 1994). In other words, this model has the structure of a generalized linear model (Knoblauch & Maloney, 2012; McCulloch & Searle, 2001) with a highly specialized link function that allows for nonlinear regression of item responses in a single-stimulus recognition task. The specialized link function is exactly such that the stimulus exposure duration *t* and the participant’s perceptual threshold *t*_0_ are both taken into account in accordance with TVA.

It was recently found that perceptual processing speed *v* is modulated by the observer’s expectation regarding the foreperiod between a cue and a subsequent target letter occurrence (Vangkilde et al., 2012; Vangkilde, Petersen & Bundesen, 2013). Specifically, in a single-letter recognition experiment (Vangkilde et al., 2012), two levels of expectancy were induced in the participants by two types of trials, one type with a higher hazard rate of stimulus presentation than the other. Across all participants perceptual processing was 40 % faster in the high expectancy condition compared with the low expectancy condition. This finding was interpreted as suggesting that higher expectations speed up perceptual processing.

However, it is well known that maintaining attention over a prolonged period of time may negatively affect attentional efficiency (Robertson et al. 1997). Even though such effects of “time-on-task” could potentially hinder optimal performance, they are rarely taken into account in studies that do not focus explicitly on sustained attention. Thus, an alternative explanation of the finding by Vangkilde et al. (2012) could be that low-expectancy trials are substantially more susceptible to time-on-task effects leading to a rapid decline in processing speed across a test session which is not seen in the high-expectancy trials.

To exemplify the explanatory power of the model expressed in Eqs. 1 and 2, we present a new analysis of the same experiment (Vangkilde et al., 2012, Experiment 3), this time including “time-on-task” as a potentially explanatory variable which is tested in the same way as variables represented in terms of trial types.

## Method

### Participants

Each of eight young female participants completed eight sessions of 480 trials each.

### Procedure

**Fig. 1:**
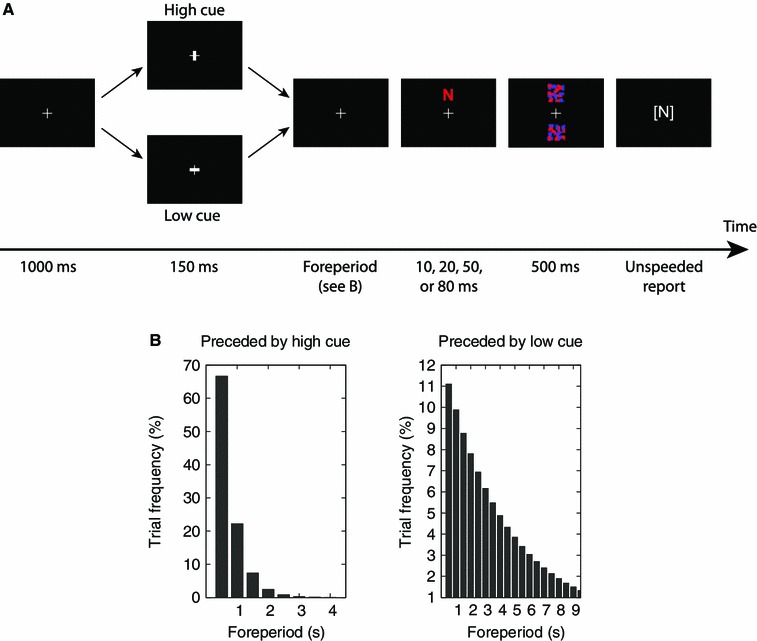
Experimental procedure. **a** Time course of a single trial. An initial fixation cross was presented. Then a brief cue appeared, to remind the participant of the hazard rate condition (high vs. low). The fixation cross then reappeared in a variable foreperiod before the *single target* letter was presented either above (as shown) or below the fixation cross before being masked. The participant then reported the letter identity if known. **b** Foreperiod distributions. These were defined to be geometric and such that, in the high hazard rate condition the expected foreperiod was 0.75 s, and in the low hazard rate condition it was 4.5 s

The events during a trial are illustrated in Fig. 1a. An initial fixation cross was presented after which a brief cue appeared to remind the participant of the hazard rate condition (high vs. low). High hazard rate was indicated by brightening of the vertical line, low hazard rate was indicated by brightening of the horizontal line. The fixation cross then reappeared in a variable foreperiod (cue-target waiting time) before the single target letter (drawn randomly from a set of 20 letter types) was presented either above or below the fixation cross before being masked. The participant then reported the letter identity, if known, via the keyboard and without time constraints. To complete the trial and continue to the next one, participants pressed the spacebar. The exposure duration *t* of the target letter was randomly sampled from the set {10 ms, 20 ms, 50 ms, 80 ms} such that all exposure durations were used equally over the course of a session.

The hazard rate (high vs. low) alternated between blocks of 60 trials. The foreperiod between the cue and the target letter was chosen at random from the set {0.5 s, 1.0 s, 1.5 s,…} following two different geometric distributions which are shown in Fig. 1b. The foreperiod distributions were defined such that, in the high hazard rate condition the expected foreperiod was 0.75 s (a hazard rate of 1.33 Hz), and in the low hazard rate condition it was 4.5 s (a hazard rate of 0.22 Hz).

### Computational model

For the computational GLIRT TVA model, the cue-target foreperiod (*FP*) of 0.5 s was chosen as the reference, so all other foreperiod coefficients were relative to this. For the hazard rate (*HR*), the low condition was the chosen reference. A time-on-task variable (*T*) was defined on the single-trial level by translation and scaling of the stimulus-onset time relative to the session such that the value of *T* increased monotonically from 0.0 on the first trial of the session to 1.0 on the last trial of the session (the 480th trial; the first trial was the reference trial).

Four nested models were considered. For any proposition *q*, let {*q*} be the binary truth value (0 or 1) of *q*. In the first model (Model 1), the natural logarithm of the perceptual processing speed of the correct categorization of the stimulus letter shown on trial *n* is given by

3 $$\begin{gathered} \ln\left( {v_{n} } \right) \, = a_{1} + a_{2} \left\{ {FP_{n} = \, 1.0 \, s} \right\} \, + a_{3} \left\{ {FP_{n} = \, 1.5 \, s} \right\} \, + a_{4} \left\{ {FP_{n} \ge \, 2.0 \, s} \right\} \hfill \\ + a_{5} \left\{ {HR_{n} = \, high} \right\} \, + a_{6} T_{n} \left\{ {HR_{n} = \, high} \right\} \, + a_{7} T_{n} \left\{ {HR_{n} = \, low} \right\} \hfill \\ \end{gathered}$$

where *a*_1_ = ln(*v*_base_), and *T*_*n*_ = (*A*_*n*_ − *A*_1_)/(*A*_480_ − *A*_1_) is the time-on-task variable, *A*_*n*_ being the onset time of trial *n*, for *n* = 1, 2,…, 480. Parameter *v*_base_ is the value of *v* in the reference condition (i.e., when *FP* = 0.5 s, *T* = 0.0, and *HR* = low). By exponentiating both sides of Eq. 3 a simple multiplicative structure is obtained,

$$\begin{gathered} v_{n} = v_{base} \times \exp\left( {a_{2} \{ FP_{n} = \, 1.0 \, s\} } \right) \times \exp\left( {a_{3} \{ FP_{n} = \, 1.5 \, s\} } \right) \times \exp\left( {a_{4} \{ FP_{n} \ge \, 2.0 \, s\} } \right) \hfill \\ \times \exp\left( {a_{5} \{ HR_{n} = \, high\} } \right) \times \exp\left( {a_{6} T_{n} \{ HR_{n} = \, high\} } \right) \times \exp\left( {a_{7} T_{n} \{ HR_{n} = \, low\} } \right), \hfill \\ \end{gathered}$$

similar to the structure of the basic rate equation of TVA (Bundesen, 1990, Eq. 1).

A sequential likelihood ratio test was designed to test Models 1–4 (i.e., effects of the foreperiod and hazard rate, as well as time-on-task effects including possible interaction with the hazard rate). Maximum-likelihood estimation of the model coefficients *a*_*j*_ in Eq. 2 was achieved via chain rules extending the Newton step (Dyrholm, Kyllingsbæk, Espeseth, et al. 2011) for estimating *v*_*n*_. Estimated model coefficients *a*_*j*_ were mapped to [exp(*a*_*j*_) − 1] × 100 % to represent the percentage difference in perceptual processing speed per unit increase of the corresponding explanatory variable *x*_*j*_. For each of the four models, the individual coefficients were tested on the group level against the null hypothesis that the percentage difference was zero. This was done for each model coefficient by summing the corresponding 64 likelihood ratio test statistics (one per subject per session). Significance levels were then derived from a Chi-square distribution with 64 degrees of freedom.

## Results

**Fig. 2:**
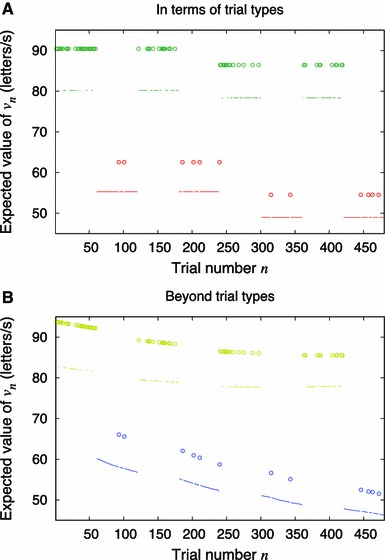
Expected value of the perceptual processing speed *v*_*n*_ given the trial types and the target onset times of an exemplary session. Model coefficients were set to the sample average. The trial types were trials with high hazard rate (*green and yellow*) versus trials with low hazard rate (*red and blue*) and trials with a foreperiod of 1 s (*circled dots*) versus trials with other foreperiods (*simple dots*). **a** The output of a conventional analysis, Model 7, where time-on-task is represented in terms of early and late trial types. **b** The output of Model 3, which differs from the conventional analysis by treating time-on-task and manipulated variables equally on the single-trial level. The divergence over trials between the results from the two hazard rate conditions (*yellow* vs. *blue*) shows very clearly the interaction between time-on-task and hazard rate

Table 1 shows the progression of the sequential likelihood ratio test which resulted in the selection of Model 3. This model contained four significant coefficients on the group level representing effects on the perceptual processing speed *v.* Averaged across participants and sessions the model is summarized as follows (cf. Table 1): An increase in *v* by 7 % when the foreperiod was 1.0 s as compared to the other foreperiods, a 28 % increase in *v* when the hazard rate was high compared to when it was low, and a gradual decrease in *v* over the course of a session amounting to 4 % in the high hazard rate condition and 27 % in the low hazard rate condition. That is, the gradual decrease in perceptual processing speed over time happened at significantly different rates in the two different hazard rate conditions (see Fig. 2). This interaction was detected in the test by rejecting Model 4 when posed as an alternative to Model 3. The modeling of this interaction using time-on-task as a single-trial measure caused a strong reduction in the estimated magnitude of the temporal expectation effect (compare Models 3 and 4 in Table 1): From an estimated 46 % increase in processing speed *v*, down to an estimated 28 % increase in *v* in the high hazard rate condition as compared with the low hazard rate condition.

**Table 1:** Testing with a single-trial measure of time-on-task

Variable	Coefficient (as % difference)			
	Model 1	Model 2	Model 3^†^	Model 4
In terms of trial types				
Foreperiod				
=1.0 s	5.24***	4.91***	7.28***	7.46**
=1.5 s	−2.21			
≥1.5 s		−5.21		
≥2.0 s	−4.46			
Hazard Rate				
=high	25.30*	24.17*	28.38***	45.59***
Beyond trial types				
Time-on-task				
*T*				−16.52***
Interactions				
*T* × {*HR* = high}	−3.93*	−4.01*	−3.73*	
*T* × {*HR* = low}	−26.46***	−26.64***	−26.68***	

Estimated differences were given by GLIRT coefficients represented as percentage change in *v* value (perceptual processing speed) per explanatory variable unit increase on average across subjects and sessions. From Model 1 onwards, the foreperiod (*FP*) coefficients were not significant beyond the *FP* of 1.0 s. Model 2 was designed as an alternative to simply eliminating the nonsignificant *FP* coefficients beyond 1.0 s. The step from Model 1 to Model 2 could not be rejected, −2ln**Λ** = 66.7, *p* [> *χ* ^2^(64)] = .383. Model 3 was designed to test elimination of *FP* coefficients beyond 1.0 s, and the step from Model 2 to Model 3 could not be rejected, −2ln**Λ** = 58.3, *p*[> *χ* ^2^(64)] = .677. Model 4 was designed to test whether the time-on-task (*T*) effects were independent of the hazard rate (*HR*) conditions, but this model was rejected in favor of Model 3, −2ln**Λ** = 92.1, *p*[> *χ* ^2^(64)] = .012. Model 3 won the model selection as further nesting to Model 4 was rejected

*HR* = hazard rate; *T* = time-on-task

^†^Model 3 wins the model selection. Further nesting to Model 4 was rejected, *p* < .05

*p < 0.05, ** *p* < 0.01, *** *p* < 0.005

Table 2 shows an almost identical test except that time-on-task is represented as a factor with two levels: early vs. late. That is, instead of treating each trial uniquely by its timestamp, two trial types have been defined as those that fall in the first half and those that fall in the second half of the experiment. The test in Table 2 concluded in agreement with the previous test that time-on-task interacts with the hazard rate condition. However, the main effect of the hazard rate manipulation was now estimated to yield a 41 % increase from the low to the high hazard rate condition.

**Table 2:** Testing time-on-task in terms of trial types

Variable	Coefficient (as % difference)			
	Model 5	Model 6	Model 7†	Model 8
In terms of trial types				
Foreperiod				
=1.0 s	5.25***	4.91***	7.16***	7.22**
=1.5 s	−2.42			
≥1.5 s		−4.97		
≥2.0 s	−4.04			
Hazard rate				
=high	37.79***	36.41***	40.75***	49.19***
Time-on-task trial type				
{*Z* = late}				−7.35
Interactions				
{*Z* = late} × {*HR* = high}	−1.24**	−1.12*	−.97**	
{*Z* = late} × {*HR* = low}	−12.49*	−12.56*	−12.72*	

Estimated differences were given by GLIRT coefficients represented as percentage change in *v* value (perceptual processing speed) per explanatory variable unit increase on average across subjects and sessions. Time-on-task is represented in terms of early and late trial types

^†^Model 7 wins the model selection. Further nesting to Model 8 was rejected, *p* < .005

*p < .05, **p < .01, ***p < .005

Compare the effect size of 41 % obtained in terms of trial types with the effect size of 28 %, which was found using time-on-task as a single-trial measure. A model selection problem arises: Which one is the better estimate? To answer this question we computed the Bayes factor per session by the ratio between marginal likelihoods as derived analytically and implemented for the single-stimulus TVA by Dyrholm, Kyllingsbæk, Espeseth, et al. (2011). With an average Bayes factor of 6.97 to one against, the single-trial model was substantially better than the trial type model (see, e.g., Rouder et al. 2012, for a contemporary description of Bayes factors).

An even worse result than the 41 % could have been obtained if one had waited to introduce the time-on-task trial types until making a post hoc check for confounding variables. This is evident from Model 8 in Table 2 where the time-on-task trial type variable is found to be insignificant. At this point a naive experimenter could have concluded incorrectly that time-on-task effects were negligible. Estimating the GLIRT model that comes out of Model 8 with the time-on-task trial type variable removed yields a main effect size of 49 % increase from the low to the high hazard rate condition—an effect size estimate which is 1.75 times higher than our current best estimate of 28 %.

## Discussion

We have presented a general method for analysis of experimental data through the use of mathematical models treating measures of potentially confounding factors and manipulated variables as equals on the single-trial level. We have also shown how the method can be applied with models that subsume under GLIRT. Specifically, we showed that the special case of TVA that is commonly used in single-item recognition is also a special case of GLIRT, and presented a thorough reanalysis of a single-letter recognition experiment (Vangkilde et al., 2012, Experiment 3) based on TVA. Our exemplary analysis incorporated a single-trial measure of time-on-task although this variable was neither manipulated nor assumed constant. Formal model selection showed that this way of estimation was more precise than the one obtained using early and late trial types. Qualitatively speaking, the model selection showed that the confounding interaction was gradual rather than reflecting a sudden change in type from early to late trials. Note that the gradual model is more general in nature than the trial type model: There are trivial scalar functions of the gradual time-on-task measure which yield the equivalent of the trial type model, but not the other way round. Naturally, one may try other nonlinear transformations of explanatory variables that go beyond trial types, thereby finding quantitatively better mathematical models of behavior (Cavanagh et al., 2011; Dyrholm et al. 2012). Our method differs from generalist data mining methods (e.g., Hinton & Salakhutdinov, 2006) by predicting through cognitive parameters. The method also differs from cognitive model-based functional neuroimaging (O’Doherty et al. 2007) by having behavioral response predictability as the explicit objective. In situations with limited data, the method should be extended to a mixed/random effects framework.

In summary, we have presented a method for checking the extent to which something measurable has an effect on observed behavioral responses. The method is readily applicable with models that fall under GLIRT by including the potentially confounding measured variables along with the manipulated variables on the single-trial level using standard tests (Mellenbergh, 1994). Our detailed example of this incorporated a measure of time-on-task in a single-letter identification response model. A measure of time-on-task will almost always be available, but a wealth of other measures may also be available depending on the paradigm, including measures of previous stimuli and responses, and physiological measures.

## Footnotes

1 In this article, a "confounder" means a variable that is a source of systematic error because it co-varies with one or more independent variables (the most traditional meaning of the word) or because it modifies the effect of some of the independent variables of interest. Note that confounders are present in almost any study.

2 Or, at least, under a modified version of GLIRT in which the link function may vary between stimuli (cf. stimulus parameter *t*) and subjects (cf. subject parameter *t*_0_).
